# Exposure to Tobacco, Environmental Tobacco Smoke and Nicotine in Pregnancy: A Pragmatic Overview of Reviews of Maternal and Child Outcomes, Effectiveness of Interventions and Barriers and Facilitators to Quitting

**DOI:** 10.3390/ijerph17062034

**Published:** 2020-03-19

**Authors:** Gillian S. Gould, Alys Havard, Ling Li Lim, The PSANZ Smoking in Pregnancy Expert Group, Ratika Kumar

**Affiliations:** 1 School of Medicine and Public Health, The University of Newcastle, Callaghan 2308, Australia; linglilim@gmail.com (L.L.L.); ratika.kumar@newcastle.edu.au (R.K.); 2 Centre for Big Data Research in Health, UNSW Sydney, Sydney NSW 2052, Australia; alys.havard@unsw.edu.au; 3 Perinatal Society of Australia and New Zealand, Mornington, Victoria 3931, Australia; psanzpositionstatementonsmokinginpregnancy@newcastle.edu.au

**Keywords:** smoking cessation, pregnancy, environmental tobacco smoke, smokeless tobacco, e-cigarettes, maternal and child health, barriers to smoking cessation, smoking cessation interventions

## Abstract

The aim of this review of reviews was to collate the latest evidence from systematic reviews about the maternal and child health outcomes of being exposed to tobacco and nicotine during pregnancy; the effectiveness of interventions designed to reduce these exposures, and barriers to and facilitators of smoking cessation during pregnancy. Two databases were searched to obtain systematic reviews published from 2010 to 2019. Pertinent data from 76 articles were summarized using a narrative synthesis (PROSPERO reference: CRD42018085896). Exposure to smoke or tobacco in other forms during pregnancy is associated with an increased risk of obstetric complications and adverse health outcomes for children exposed in-utero. Counselling interventions are modestly effective, while incentive-based interventions appear to substantially increase smoking cessation. Nicotine replacement therapy is effective during pregnancy but the evidence is not conclusive. Predictors and barriers to smoking cessation in pregnancy are also discussed. Smoking during pregnancy poses substantial risk to mother’s and child’s health. Psychosocial interventions and nicotine replacement therapy (NRT) appear to be effective in helping pregnant women quit smoking. Barriers to smoking cessation must be identified and steps taken to eradicate them in order to reduce smoking among pregnant women. More research is needed on smoking cessation medications and e-cigarettes.

## 1. Introduction

Smoking in pregnancy constitutes the largest remediable risk factor for maternal and child health. A multitude of health effects have been documented [1]. For pregnant woman these include increased risks of obstetric complications, e.g., higher rates of spontaneous abortions, ectopic pregnancies, placental abruption, placenta praevia, premature labour, and preterm birth, compared with pregnant non-smokers [1]. Previous observational research has found that smoking by the mother during pregnancy leads to risks for the unborn baby, such as increase in a risk of stillbirth, low birth weight (LBW) and small for gestational age (SGA) compared to babies born to women who do not smoke during pregnancy [2,3,4]. Prenatal maternal smoking is also associated with risk of sudden unexplained death in infancy (SUDI) [5]. Into childhood, offspring may experience increased risks of respiratory problems, cancers, neurodevelopmental and behavioral problems, as well as increased long-term risks of non-communicable diseases. 

Smoking cessation has a positive impact on the trajectory of these health problems, especially if achieved within the first 20 weeks of gestation [6]. However, significant challenges may influence women being able to achieve abstinence from smoking once becoming pregnant. Barriers include social environments that encourage smoking, stressors, mental health issues, interpersonal violence, substance use and lack of access to suitable antenatal and smoking cessation services. Health providers face their own challenges to provide evidence-based smoking cessation care such as low knowledge and confidence about effective smoking cessation treatments, fear that their advice may adversely influence their relationship with a pregnant woman, and lack of optimism for successful treatment outcomes [7,8,9].

This pragmatic overview of reviews was initiated as a part of developing an evidence informed Position Statement focused on supporting women to stop smoking in pregnancy for the Perinatal Society of Australia and New Zealand (PSANZ). An overview of reviews methodology was an appropriate and resource-efficient alternative to a conventional systematic review, considering the large volume of primary studies and existing previous systematic reviews on the topic of tobacco use in pregnancy. Although much is known about the effects of smoking in pregnancy, there have been few overviews of this scale bringing together top-level evidence. The aim of this paper is to review and summarize the latest literature related to tobacco use in pregnancy including the effects of tobacco on mother and baby, the effects of other tobacco products and electronic nicotine delivery systems (ENDS) and secondhand smoke exposure in pregnancy, interventions to promote cessation of tobacco use in pregnancy, barriers and predictors of smoking and smoking cessation in pregnancy, and interventions to prevent or treat tobacco use in pregnancy. The effects from smoking were considered for the pregnant woman and babies up to two years of age.

## 2. Materials and Methods 

This overview of reviews was originally developed by a PSANZ working group made up of ten experts (Gillian Gould, Ling Li Lim, Vicki Flenady, Alison Goodfellow, Alys Havard, Nusrat Homaira, Phillippa Middleton, Lynn Sinclair, Susanne Wooderson, Sarah Jane Perkes) in smoking cessation, perinatal health, paediatric health and epidemiology. The working group was established to develop a position statement on smoking during pregnancy for PSANZ. The review protocol was registered in 2017 with PROSPERO (International Prospective Register of Systematic Reviews) with reference number CRD42018085896. Updating of searches was done in 2019 for the purpose of the present review.

### 2.1. Research Questions

This review endeavoured to answer three broad questions: 

1. What are the maternal and child health outcomes of being exposed to active and passive tobacco smoke and other tobacco or nicotine products during pregnancy?

2. What interventions are effective for helping women stop using tobacco or other nicotine products during pregnancy and stop or reduce their exposure to environmental tobacco smoke?

3. What are the predictors of continuing and discontinuing smoking or active and passive exposure to tobacco and nicotine products among pregnant women? 

For the purpose of this review, we systematically synthesized research from existing systematic reviews and meta-analyses to answer the aforementioned research questions. This included identifying and summarizing key concepts and evidence related to health outcomes, effectiveness of interventions, predictors of health behaviors and gaps in the research pertinent to practice, policymaking, and research [10].

### 2.2. Literature Search and Search Strategy

A search strategy was developed by the PSANZ team of experts in consultation with a research librarian after examining a sample of relevant systematic reviews for the common terminologies utilized in these reviews. The search strategy so developed was also agreed upon by all the co-authors. The search strategy was tailored for each database to get the most relevant results. Two electronic databases namely PubMed and CINAHL (Cumulative Index to Nursing and Allied Health Literature) were searched using the search terms for tobacco exposure, i.e., ‘smoking’, ‘tobacco’, ‘cigarette’, ‘nicotine’, ‘e-cigarette’, ‘electronic cigarette’, ‘vaping’, ‘second-hand smoke’, ‘secondhand smoke’, ‘environmental tobacco smoke’, ‘smokeless tobacco’, ‘smoke-less tobacco’ and ‘passive smoking’ in conjunction with pregnancy related search terms ‘pregnancy’, ‘pregnant’, ‘prenatal’ and ‘maternal’. Searches were limited to peer reviewed, English language systematic reviews, meta-analyses and meta-syntheses published between January 2010 and December 2019. To ensure that the most relevant reviews were identified reference lists were reviewed for further suitable reviews. Initially, databases were searched by LLL with support from University of Newcastle Senior Research Librarian up until the 26/04/2017. A second search was carried out by RK till December 2019 to include the latest systematic reviews relevant to the topic.

### 2.3. Screening and Data Extraction 

All citations were imported into the Microsoft Windows based bibliographic manager Endnote X8.2 to create a composite library and duplicate citations were removed. First level screening was done by RK, LLL and AH whereby titles and abstracts of the articles were read and excluded if they did not meet the eligibility criteria. Full texts of all articles deemed relevant after first level screening were acquired for more detailed review. Second level screening of the full texts was done by LLL and RK where all articles were read in full and checked against the eligibility criteria. Disagreements at both levels were resolved by discussion among the authors and where relevant the PSANZ expert team. Data from eligible articles was extracted by RK and LLL into a Microsoft Excel data extraction form under the headings: study number, author, year of publication, title, source document, included studies, populations, interventions, outcomes measured and overall results and effects. 

### 2.4. Inclusion and Exclusion Criteria

This overview of reviews included systematic reviews, meta-analyses, integrative or comprehensive reviews if they had a methodology which included: inclusion and/or exclusion criteria for studies, in the English language and were published between 2010 to end of December 2019. Reviews were assessed as suitable if they contained smoking/tobacco as one of the inclusion criteria. Reviews of reviews were excluded. 

Publications about women who were pregnant and also smoked tobacco, smoked tobacco with cannabis, used smokeless tobacco products, e-cigarettes or were exposed to second-hand smoke during pregnancy were included in this review. We also included papers about effects of smoking during pregnancy on offspring. Childhood outcomes for 0–2 years of age were included and where childhood outcomes were described over a wider age range, we included the review if 0–2 years was reported as a separate variable or if the condition in focus is normally diagnosed before 2 years of age. 

Interventions to reduce tobacco smoking consumption during pregnancy were included in this review. Both interventions offered alone or in combination were included. 

### 2.5. Types of Outcome Measures

- Neonatal, infant, and child outcomes (0–2 years age) of mothers who smoked during pregnancy;
- Maternal obstetric outcomes;
- Smoking cessation (self-reported and bio-chemically validated);
- Prevention of second-hand smoke exposure of pregnant women;
- Barriers and facilitators to smoking cessation;
- Studies were excluded if papers lacked a systematic methodology as above, were primary or empirical studies or were animal studies.

### 2.6. Quality Assessment 

Quality assessment was not conducted, as this was a pragmatic overview and it was not the intention to exclude reviews based on quality ratings. 

### 2.7. Data Synthesis 

Since the review aimed to incorporate a wide array of themes related to tobacco use in pregnancy, each member of the PSANZ team of experts was entrusted with reviewing and preparing a summary of their allotted topic. A narrative synthesis was prepared by RK and GG along with individual (or paired) collaborators on their respective topics. 

RK refreshed the search and synthesized information from all literature included from the updated search from April 2017 onwards.

## 3. Results

**Figure 1:**
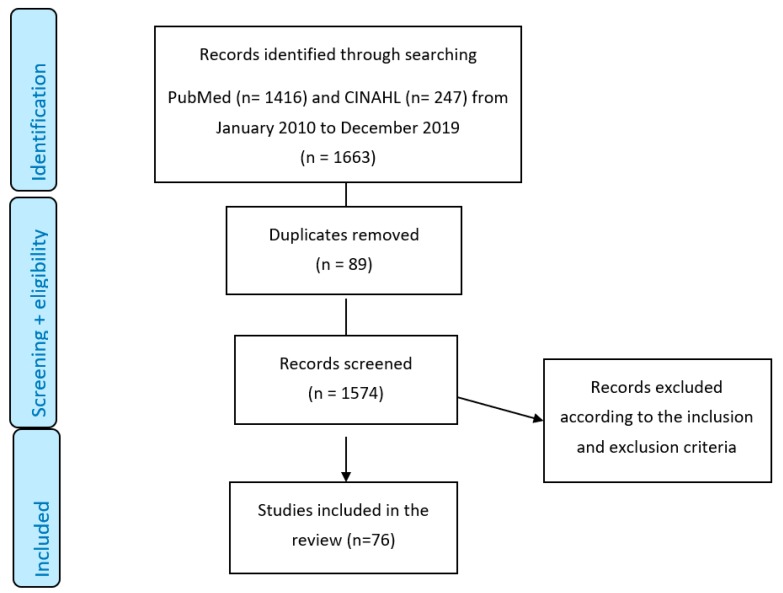
PRISMA flow diagram.

The contents of this overview of review are based on 76 included systematic review papers. See Figure 1 for the Preferred Reporting Items for Systematic Reviews and Meta-Analyses (PRISMA) flow chart. Some reviews covered more than one area of interest.

### 3.1. Risks of Tobacco Exposure during Pregnancy 

Risks are summarized separately for pregnant women and the foetus or child up to two years of age in Table 1 and Table 2 respectively. 

#### 3.1.1. Risks of Smoking for Pregnant Women 

Seven papers were included on pregnancy and birth complications for mothers who smoked during pregnancy [11,12,13,14,15,16,17].

Birth complications: Women who smoked during pregnancy have an increased risk of obstetric complications, compromised psychological health and micronutrient deficiencies. Exposure to tobacco constituents in early pregnancy likely affects placental development directly or indirectly by reducing blood flow, which creates a pathologically hypoxic environment [41]. Shobeiri et al. (2017), in two different meta-analyses, found that women who smoked during pregnancy were more likely to experience placental abruption [11] (odds ratio (OR) 1.80, 95% confidence interval (CI) 1.75, 1.85 and relative risk (RR) ratio: 1.65, 95% CI 1.51–1.80) and placenta previa [12] (OR 1.42, 95% CI 1.30–1.54 and RR 1.27, 95% CI: 1.18–1.35) compared to women who did not smoke during pregnancy. 

Problems with assisted reproductive technology: Two separate reviews reported pregnancy outcomes among women who were using assisted reproductive technology (ART). Budani (2018) reported a significant decrease in the live birth rate per cycle (OR 0.59, 95% CI 0.44–0.79), a significantly lower clinical pregnancy rate per cycle (OR 0.53, 95% CI 0.41–0.68), and a significantly increased spontaneous miscarriage rate (OR 2.22, 95% CI 1.10–4.48) for women who smoked. [16] Similarly, Purewal (2019) found that women who did not smoke undergoing ART were significantly more likely to achieve a live birth or pregnancy than women who did smoke (OR 1.46, 95% CI: 1.228–1.727) [17].

Miscellaneous complications: There was preliminary evidence that women who persistently smoked in pregnancy experienced elevated stress levels as measured by subjective self-report measures and objective measures such as hair cortisol levels. [14] This review showed that there is a significant positive association between stress measures or the existence of stressors and the presence of smoking behaviors among pregnant women [14]. Smoking during pregnancy was associated with changes in nutrient levels in pregnant women such as a decreased folate and vitamin B12 levels and increased homocysteine levels [15]. In contrast, the rates of some pregnancy complications have been found to be lower in women who smoked during pregnancy than those who did not smoke, for example, hyperemesis gravidarum (OR 0.40, 95% CI: 0.24–0.56) [13]. 

#### 3.1.2. Risks for Foetus and Child

Twenty six papers were included on risks of maternal smoking for babies and children [3,4,5,18,19,20,21,22,23,24,25,26,27,28,29,30,31,32,33,34,35,36,37,38,39,40]. Maternal smoking in pregnancy was associated with a myriad of adverse health effects for the unborn baby and in the first two years of life of the offspring. 

Development and birth defects: Foeti of mothers who smoked during pregnancy were recorded to have reduced foetal measurements including head size, femur length, foetal weight and transcerebellar diameter [34,35,37]. Exposure to tobacco during pregnancy has consistently been reported to result in low birth weight (LBW) [42,43,44,45,46]. Smoking tobacco during pregnancy can increase the chances of LBW by 200% (OR = 2.00, 95% CI: 1.77–2.26) [33]. Smoking was associated with a 10–30% increased risk of congenital birth defects including cardiovascular, digestive, musculoskeletal and face and neck defects, with a dose-response relationship [20,22]. The risk of spina bifida increased by over 50% for newborns whose mothers smoked during pregnancy (OR 1.55, 95 % CI = 1.06–2.26) [23]. Mothers who smoked during pregnancy may also have an 18% greater chance of having a male child with cryptorchidism than mothers who did not smoke [28,40]. There was approximately a 36% greater odds of babies being born with a cleft lip or palate if the mother smoked during pregnancy [29]. Smoking during pregnancy is a modest risk factor for heart defects such as atrial and ventricular septal defects [32].

Infants’ mortality: Adverse newborn health outcomes associated with maternal smoking during pregnancy included a ~50% increase in the risk of stillbirth, [4,30] a 22% increase in the risk of neonatal death [30] and a 33% increase in the risk of perinatal death [30]. Compared to controls, the odds of mothers smoking (prenatally or postnatally) were 200% higher in children who died due to SUDI [5]. The relationship between SUDI and maternal smoking was dose dependent and risks increased significantly if the infant co-slept with mothers who continued smoking postnatally [5]. 

Childhood cancers: Adverse outcomes in children included an modestly elevated risk of childhood cancers including non-Hodgkin’s lymphoma (OR = 1.22, 95% CI = 1.03–1.45) [18] and acute lymphoblastic leukaemia (OR = 1.10, 95%CI = 1.02–1.19) [31]. There was also a 20% greater odds (OR 1.22; 95% CI 1.04 to 1.44) of developing neuroblastoma during childhood, if mothers smoked during pregnancy [38].

Other health outcomes in infants: Smoking during pregnancy also predicts poor respiratory outcomes. Burke (2011) reported that prenatal (OR = 1.41, 95% CI = 1.20–1.67) and postnatal maternal smoking (OR = 1.70, 95% CI = 1.24–2.35) increased the risk of wheezing in children aged ≤2 years, with wheezing being the most common cause of acute hospital presentations. Maternal smoking during and after pregnancy and household exposure to passive smoking were strong risk factors associated with wheeze and respiratory tract infections in children aged ≤2 years old, including bronchiolitis, pneumonia, bronchitis, pulmonary tuberculosis, and otitis media [2,19,26,47]. Maternal smoking during pregnancy was associated with a significantly increased risk of asthma, however, the effect of postnatal smoking and household exposure to smoking on the development of asthma, remains unclear [19]. Smoking in pregnancy was an independent risk factor for visual impairments including strabismus, refractive errors and retinopathy [24]. Smoking in pregnancy was not associated with autism spectrum disorder [27]. At epigenetic level, tobacco smoking during pregnancy can change DNA methylation and miRNA expression in the placental tissue. These in turn can lead to changes in gene expressions which may affect the development of health conditions in offspring [39].

### 3.2. Risks Associated with Exposure to Tobacco and Nicotine in Other Forms, E-Cigarettes, and Second-Hand Smoke

Eleven reviews (Table 3) described the health effects of second-hand smoke exposure during pregnancy [19,26,36,42,45,48,49,50,51,52,53], three reviews (Table 3) described health effects of using smokeless tobacco during pregnancy [43,44,54], while two reviews (Table 3) were about health outcomes of water pipe (shisha) use during pregnancy [46,55]. 

Second-hand smoke: Tobacco use poses serious health risks not only for the individual smoker but also for non-smokers due to second-hand smoke (SHS) exposure, especially children and pregnant women. Despite restrictions to smoking in public places, pregnant women may be exposed to SHS in the home, especially in populations with higher smoking prevalence such as in Indigenous communities. 

Risks to mothers: Women who were exposed to SHS were at a 20% greater risk of giving birth prematurely [52]. Although there was only limited evidence, SHS smoke exposure in pregnant women has been linked to a 70% increase in the risk of mental health problems such as depression and suicide ideation [53].

Risks to babies: A meta-analysis of 19 observational studies indicated that women who did not smoke, but were exposed to SHS during pregnancy, had an increased risk of still birth of 23% and of congenital malformations of 13%, although SHS did not seem to increase the risk of spontaneous abortions [48]. Another meta-analysis found smaller head circumference (weighted mean difference—0.11 cm; 95% CI—0.22 to 0.01 cm) and lower birthweight (weighted mean difference—60 g, 95% CI—80 to –39 g) among infants exposed to SHS in-utero [42]. There was greater risk of discontinuation of breastfeeding before six months among women exposed to SHS during pregnancy (OR 1.07, 95%CI: 1.01–1.14) [50]. SHS exposure at home (paternal of maternal) increased the risk of wheeze in children aged ≤2 years by ~30% (OR = 1.35, 95% CI = 1.10–1.64) [19,26]. Having any household member who smoked increased the chances of children ≤2 years getting lower respiratory infection by more than 50% (OR 1.54, 95% CI 1.40 to 1.69) [49]. The strongest effect was for bronchiolitis, where the risk increased by more than 250% (OR 2.51, 95% CI 1.96 to 3.21) [49]. Postnatal maternal smoking (exposing the infant to SHS) carried double the risk of lower respiratory infection compared to prenatal smoking (OR 1.58 vs. 1.24) [49]. Similar to active smoking by pregnant mothers, the risk of orofacial clefts in infants was also accentuated by approximately 200% when mothers were exposed to passive smoking during pregnancy [51]. Babies exposed to passive smoking also have increased odds of neural tube defect (OR 1.898; 95% CI 1.557–2.313) [36]. 

Waterpipe and smokeless tobacco use: Waterpipe smoking by pregnant women was associated with LBW, low Apgar score, pulmonary complications at birth, and infant mortality [46,55]. Smokeless tobacco (e.g., chewed or oral snuff) delivers high levels of nicotine that may cause dependence, and in pregnancy increased rates of stillbirth, preterm birth, small for gestational age, LBW, and altered male to female live birth ratio [43,44,54]. Suliankatchi (2016) found that women who used smokeless tobacco during pregnancy were almost at double the risk of giving birth to babies with LBW (OR = 1.88, 95 % CI 1.38–2.54) and were at a significantly greater risk of birthing babies who were preterm (OR = 1.39, 95 % CI 1.01, 1.91) or were stillborn (OR = 2.85. 95 % CI 1.62–5.01) [43].

E-cigarettes (ENDS): The search did not retrieve any systematic reviews about the use of E-cigarettes during pregnancy, (or for that matter any empirical studies, although empirical studies were not a prime focus of the search). 

### 3.3. Interventions to Reduce Tobacco Exposure in Pregnancy 

Ten papers (Table 4) described the effectiveness of interventions for smoking cessation among pregnant women [47,56,57,58,59,60,61,62,63,64]. Interventions included behavioral interventions such as contingency management (incentive-based treatment such as financial rewards), counselling, health education, social support, culturally tailored interventions and bio-feedback during ultrasound and pharmacotherapy for smoking cessation such as bupropion and nicotine replacement therapy. Interventions were offered alone or in combination. 

#### 3.3.1. Behavioral Interventions 

Akerman (2015) reported results from studies of smoking cessation interventional studies among women with opioid use disorder. Despite the high prevalence of smoking and difficulties in quitting among this population, only three studies were found to be suitable to be included in their review. Contingency management, an incentive-based treatment, was found to be the most promising intervention in one of the included studies: 48% of women were reported to have achieved a 75% reduction in smoking at 12 weeks compared to 0% in a non-contingent behavior incentive group and 2% in a group receiving usual care. Among the group receiving contingency management 31% achieved abstinence at 12 weeks [56]. Similarly, incentives were found to be twice as effective at achieving smoking abstinence as non-contingent interventions (RR 2.36, 95% CI 1.36 to 4.09) in global Cochrane review [60]. 

Counselling was generally found to be an effective smoking cessation intervention among pregnant women. Chamberlain et al., in their 2017 Cochrane review that included 102 trials, evaluated interventions such as counselling, health education, feedback, incentives, social support, exercise and dissemination [60]. High quality evidence from this review suggests that counselling increases smoking cessation in late pregnancy compared with usual care (RR 1.44, 95% CI 1.19 to 1.73), however, it was unclear if counselling prevents relapse. Feedback (information about the fetal health status or measurement of by-products of tobacco smoking to the mother; e.g., carbon monoxide monitoring, ultrasound monitoring of foetus) was found to be highly effective when provided in conjunction with counselling (RR 4.39, 95% CI 1.89 to 10.21) but less so with other types of interventions of lower intensity. However, a review conducted by Filion et al., which included 8 studies and tested counselling against usual care, suggested that standalone smoking cessation counselling and brief advice (including telephone counselling) did not seem to have a large effect on smoking cessation outcomes among pregnant women [57]. When compared to usual care, there was about a 4% absolute difference in the biochemically verified abstinence rates in favour of the smoking cessation counselling [57]. 

Racial and ethnic minorities may benefit from psychosocial interventions such as counselling, brief advice and NRT [62]. However, in a review of culturally tailored smoking cessation interventions targeted at pregnant Indigenous women, Passey et al. included two studies; neither study showed any significant effect compared to the control [61]. 

Effect of partner support on smoking cessation among pregnant women was inconsistent. Partner support was evaluated by Hemsing et al. in their review of nine studies [59]. Enhancing partner support was not found to be effective in two-thirds of the studies with only one study finding significant effect from using a video, a booklet and 10-min counselling session during two visits and a similar intervention for the partner. Seven of the nine studies reviewed (77.7%) did not find an effect from the interventions on partner smoking cessation, however there was some evidence that such interventions may improve the rate of quit attempts among the partners [59]. Similarly, Arden-Close (2017) who studied health behavior change interventions directed at couples, did not find any benefit from psychosocial interventions such as brief advice, video or informational booklet on smoking cessation outcomes in pregnant women [63]. 

#### 3.3.2. Pharmacological Interventions 

In the general population, NRT, bupropion and varenicline are the three evidence-based pharmacotherapies used for smoking cessation. In a fixed effect meta-analysis including seven studies (six of which used NRT and one bupropion) involving 1386 participants, it was found that pharmacotherapy is about twice (RR 1.80; 95% CI 1.32–2.44) as effective as usual care for smoking cessation among pregnant women [58]. 

Nicotine replacement therapy: Among all the smoking cessation pharmacotherapies, NRT has been tested most extensively among pregnant smokers. Findings regarding whether NRT use during pregnancy improved smoking cessation rates or birth outcomes have been mixed [64]. Myung et al. found that nicotine patch was 1.6 times (RR 1.60 95% CI 1.05–2.43) and nicotine gum was 1.21 times (RR 1.21, 95% CI 0.64–2.29) as effective as no treatment [58]. The most contemporary and comprehensive review of NRT to date found weak evidence to suggest that NRT was effective in promoting smoking cessation [64]. In this Cochrane meta-analysis, NRT increased smoking cessation rates by 40% (RR 1.41, 95% CI 1.03–1.93). Analysis of only placebo-controlled studies resulted in a lower cessation rate (RR 1.28, 95% CI 0.99–1.66). There was no evidence that NRT had either a positive or negative impact on outcomes from pregnancy and birth in women or babies from nine randomized controlled trials (RCTs) using NRT vs placebo including >2000 women [64]. 

Both behavioral and pharmacological smoking cessation interventions and their combinations have been found to be cost-effective. Jones (2015) reviewed economic evaluations of smoking cessation interventions across 18 studies. Seventeen out of eighteen studies reviewed suggested that cessation interventions (counselling, NRT and self-help material compared to usual care) may generally be cost-effective. Cessation interventions that involve physical activity were dominant, i.e., these interventions cost less and were more effective than usual care, although this conclusion was based on only one study, and should be interpreted cautiously [47]. 

Burpopion and varenicline: There is lack of evidence to draw a conclusion regarding the effectiveness of bupropion. All the RCTs included in the review by Myung et al. tested NRT except one which tested bupropion as an intervention and found it to be three times (RR 3.33, 95% CI 1.06–10.49) as effective as the control (no treatment) [58]. Coleman et al. in their review (one study) did not find bupropion to be an effective smoking cessation aid for pregnant women [64]. There was no systematic review evidence about the effects of varenicline during pregnancy (another medication designed to assist with smoking cessation, however not recommended for use in pregnancy) [64].

#### 3.3.3. Interventions to Reduce SHS 

Three studies (Table 4) described interventions directed at reducing SHS for pregnant women [65,66,67]. Behavior change interventions were effective in reducing second-hand smoke exposure of mothers and infants [66,67]. Behavioral interventions such as counselling, informational booklets, videos, telephone, and Quitline counselling focused on pregnant women and their partners, can result in quit rates of partners of 16%–23% in the intervention group compared the controls. [65] Interventions as simple as offering to bring partners into a consultation resulted in lower rates of indoor smoking [65]. However, there were very few studies that used objective measures such as biochemical verification of SHS exposure and hence the results of these studies using self-reported measures may not be reliable [67].

### 3.4. Predictors of Smoking and Barriers to Cessation

Fifteen papers (Table 5) were included on predictors of smoking or barriers to cessation for women [68,69,70,71,72,73,74,75,76,77,78,79,80,81,82]. Barriers and predictors of smoking cessation during pregnancy can be broadly divided into factors operating at the health provider (HP) level, social level and individual level. 

Health provider level barriers and predictors: Smoking cessation support from the health provider (HP) may be a crucial predictor of smoking cessation among pregnant women [68]. Appropriate quit smoking advice, using protocols and guidelines, following up with clients and being confident in their smoking cessation skills may predict a greater uptake of smoking cessation services by pregnant women [68]. Lack of training, time and resources on the part of HPs may reduce the consistent delivery of quit smoking advice [68]. Other provider specific barriers include a lack of knowledge regarding patient counselling and referral into treatment, low confidence in personal intervention skills, low confidence in using NRT for pregnant women, perceptions that HP advice cannot influence a patient’s behavior, that tobacco dependence treatment is not the role of HPs working with pregnant women, smoking cessation interventions for pregnant smokers are ineffective and that advising pregnant smokers to quit could be detrimental to the HP’s relationship with the patient [70,81]. HPs also reported experiencing barriers such as a lack of smoking cessation training, not receiving any reimbursement for providing smoking cessation services and no guidelines and protocols for carrying out a smoking cessation intervention [70]. Thus, barriers that HPs face may ultimately translate into non-provision of adequate smoking cessation interventions for pregnant women. Harris et al. (2019) in their review found that for Aboriginal and Torres Strait islander women, information and advice regarding potential adverse effects of smoking on the foetus, or lack thereof, from health professionals either facilitated cessation of smoking in pregnancy or was a barrier to quitting [81]. Health risk messages about smoking experienced by Indigenous women often are non-salient or lack appeal and may fail to engage Australian Indigenous communities [71]. 

Social and Individual level predictors and barriers: Individual level predictors of continued smoking in pregnancy are as follows: multiparity, older mothers, low socio-economic status, a partner who smokes and nicotine dependence [73,82]. Riaz (2018) included a total of 15 clinical trials and 40 observational studies to review the predictors of smoking cessation in pregnancy. According to their review, the following predictors almost doubled the odds of smoking cessation in pregnant women: a higher level of education (OR 2.16, 95% CI 1.80–2.84), higher socio-economic status (OR 1.97 ; 95% CI 1.20–3.24), overseas maternal birth (OR 2.00; 95% CI 1.40–2.84), not consuming alcohol before and/or during pregnancy (2.03 (1.47–2.80), primiparity (OR 1.85; 95% CI 1.68–2.05), planned breastfeeding (OR 1.99; 95% CI 1.94–2.05), perceived adequate pre-natal care (OR 1.74; 95% CI 1.38–2.19) and no depression (OR 2.65; 95% CI 1.62–4.30) [79]. Other significant predictors were having a Medicaid coverage (in the United States) or private insurance (OR 1.54 95% CI 1.29–1.85), living with partner or married: OR 1.49 ; 95% CI1.38–1.61), partner or other members of the household do not smoke: 0.42 (0.35–0.50), a lower heaviness of smoking index score: (OR 0.45; 95% CI 0.27–0.77), a lower baseline cotinine level: (OR 0.78; 95% CI 0.64–0.94), low exposure to secondhand smoking: (OR 0.45; 95% CI 0.20–1.02), and low stress during pregnancy: (OR 0.58; 95% CI 0.44–0.77) [79].

Crane et al., (2013) in their review of research examining the relationship between intimate partner violence (IPV) and smoking, found that there was a moderate association between IPV and smoking among pregnant women (d = 0.49, k = 18 ; 95% CI = 0.38–0.59). [72] Rhodes-Keefe et al. (2015), who studied the influence of depression on rural pregnant women’s smoking found that depression may be a significant risk factor for smoking in this population. Depression and limited support may promote continuance of smoking [77]. Flemming conducted two systematic reviews that explored the barriers and facilitators of smoking cessation among pregnant women [73,75]. Flemming (2013) identified four lines of argument to trace the journeys made by women who were smokers at the start of their pregnancy, namely: (1) being a smoker, (2) being a pregnant smoker, (3) quitting and trying to quit smoking, and (4) continuing to smoke. [73] Wanting to protect their baby from harmful effects of smoking and the desire to acquire the moral identity of a non-smoker facilitated quit attempts [73]. On the other hand, difficult life circumstances, persisting disadvantage, stress and addiction thwarted quit attempts [73]. Partners smoking behaviors such as smoking in pregnant women’s presence, offering them cigarettes and putting the entire onus of quitting smoking on the mother for the sake of child’s health was a major barrier to quitting smoking [73]. Smoking in the wider social circle along with financial and psychosocial stress increased their reliance on smoking [73]. Often pregnancy itself acted as a barrier to cessation because women wanted to retain smoking as a pleasurable pastime while their other activities, such as socializing and employment, were restricted due to their pregnancy [73].

The more recent review by Flemming et al. identified four recurring themes about pregnant women’s experiences and perceptions of smoking [75]. Smoking cessation was difficult as those who smoked reported that barriers to quitting were built into their domestic, social and working lives [75]. Further barriers to quitting lay in smoking being a source of enjoyment and an addiction from which it was difficult to escape. In addition, smoking may be viewed as a way to maintain emotional stability and manage stress by pregnant women. Scepticism towards the harms of smoking (e.g., ‘lack of hard proof and hard facts’) for the baby was also a barrier to quitting [75]. This scepticism was mainly due to a lack of information on how, smoking damaged the unborn child and people’s health. However, wanting to protect their baby from harmful effects of smoking and the desire to acquire the moral identity of a non-smoker may facilitate quit attempts in pregnancy [75].

Bauld et al. reviewed 55 studies and found that partners’ support and the role of smoking within the relationship were important to quitting [78]. Graham et al. highlighted how complex circumstances during pregnancy acted as a barrier to quitting smoking [76]. Pregnancy often signified a change in employment patterns, family relationships and housing arrangements resulting in stress which made it difficult to quit [76]. Cutting down consumption was often preferred to abruptly quitting smoking without using behavioral or pharmacological therapies. Although, cutting down may be considered a method of harm reduction, it may often prevent women from quitting smoking completely [76]. 

Among Indigenous pregnant women, barriers to smoking cessation may include considering smoking a way of life and which helps one get through the day [71]. Small (2018) in their review found that being pregnant, perceived societal restrictions on smoking during pregnancy, aversion to taste and smell of smoking during pregnancy and concern for baby’s health could motivate Indigenous women to quit [80]. Women often continued to smoke during pregnancy due to higher nicotine dependency, living in a smoking environment, a lack of social support, experiencing stress, not knowing the harms of smoking or lack of impactful smoking cessation messages [80]. Smoking cessation could be facilitated among Indigenous women by making health information, interventions and programs for smoking cessation easily accessible and providing ongoing support to quit [80].

## 4. Discussion

This systematic review of reviews confirms that there is an overwhelming amount of evidence that warrants supporting women to stop smoking during pregnancy. While we focused on health problems of babies exposed to tobacco products in the first two years of life, health problems range from short to long term and may affect children’s physical and mental health throughout their life course [83,84]. 

Results obtained from this overview have some similarities to three other overviews of reviews detailed below, but their scope was more limited [2,85,86]. Zhou et al., did a comprehensive review on the adverse effects of SHS exposure, waterpipe smoking and smokeless tobacco use in pregnancy. They found that SHS may results in sudden infant death syndrome, low birthweight, decreased head circumference, respiratory infections, otitis media, asthma, childhood cancer, hearing loss, dental caries, and metabolic syndrome. Adverse cognitive and behavioral outcomes were also associated with SHS [2]. They found that waterpipe smoking among pregnant mothers may result in their children being born with low Apgar scores, birth defects (such as cardiac and hip anomalies and hydrocephalus), perinatal complications (e.g., jaundice, respiratory difficulties, and prolapse of the cord) as well as significant increases in infant respiratory distress. [2] Zhou et al. found that smokeless tobacco use during pregnancy can result in increased rates of foetal morbidity and mortality [2]. Cessation support for pregnant women should therefore extend to these other exposures. 

In Australia, 20% to 30% of women quit smoking after they become pregnant, but about half of these women relapse within six months of their delivery [87]. Predictors of maternal smoking include the social determinants of health as well as physiological changes occurring during pregnancy [88]. Lower socio-economic status combined with the high addiction liability of nicotine, means that smoking can play an important role as a tool to cope with significant financial and interpersonal stressors [89]. Additionally, as nicotine metabolism is higher during pregnancy, higher doses of NRT and greater psychological support may be required during pregnancy to control withdrawal symptoms and achieve abstinence [90]. Contingency management interventions appeared to be highly effective for smoking cessation among pregnant women, however, their sustainability depends on government smoking cessation schemes that incorporate incentives for quitting smoking. Promising interventions for smoking cessation in pregnancy include: incentive-based treatment, behavioral support counselling and pharmacotherapy [91,92,93]. Our results relating to interventions are similar to those acknowledged in another overview including 93 reviews and primary studies by Meernik et al., who found that incentive based and multicomponent psychosocial interventions were most successful for smoking cessation among pregnant women [85]. Although the level of effectiveness of NRT use during pregnancy remains unclear, perhaps due to the lower than optimal doses being trialed. NRT does not appear to have negative health impacts on mother or baby [94]. NRT use during pregnancy should be considered for women in whom behavioral interventions alone have not been successful. Despite prescription medications such as varenicline and bupropion being two to three times more effective for smoking cessation among the general population, their efficacy and safety has not been adequately assessed in pregnant women [89]. A recent Cochrane review found no significant positive or negative effects associated with the use of bupropion or varenicline in pregnancy [95]. Hence, more research on the effectiveness and safety of different pharmacological interventions, and different dosage regimes, during pregnancy are needed given promising initial results and evidence that they are the most effective smoking cessation strategy in the non-pregnant population.

The most promising interventions will not be effective if they are not implemented by health providers. Our findings confirm findings in an overview of three reviews performed by Morgan et al. who concluded that barriers to quitting smoking are complex and embedded into the individual, social and systemic milieu of a pregnant women’s day to day life [86]. Lack of assistance from health providers, smoking environments, conflicting information about ill effects of smoking, high physical and emotional dependence on smoking and physical and mental stresses associated with pregnancy all contribute to continued smoking among pregnant women [86]. A major finding of this review is that health providers also require more support to deliver culturally appropriate, evidence-based smoking cessation care during pregnancy. Important provider-specific and system/organizational barriers need to be addressed to promote health providers’ engagement in smoking cessation with pregnant smokers [70]. Service-level systems such as smoking cessation guidelines, follow-up protocols and training for health providers are associated with a greater uptake of smoking cessation services by pregnant women [68]. The implications for developing training materials for health providers are to focus on increasing knowledge on prescribing NRT in pregnancy, counselling and behavior change techniques, referral pathways to tobacco treatment specialists and understanding that women want more support to stop smoking from their health providers. 

Although a controversial topic in tobacco control [96], there was no systematic review evidence on the effect of e-cigarettes in pregnancy, due to a lack of empirical studies. In the US, prevalence of e-cigarette use among pregnant women is 4.9% to 15% [97]. Some evidence suggests that e-cigarettes are a safe alternative to tobacco smoking and can be used a as a smoking cessation aid, however the present research is inconclusive, especially in pregnancy, where there is no research yet to support or condemn their use [98]. While probably less dangerous than combustible cigarettes in non-pregnant users, the risk of harm from e-cigarettes is unclear in pregnancy because the majority of pregnancy relevant research is conducted in animal models [99,100]. More original research evaluating the outcomes associated with the use of e-cigarettes during pregnancy is needed, considering their popularity with young women [101] and potential use as a smoking cessation aid, accompanied by strong market regulation, as modelled in the United Kingdom [102].

### Strengths and Limitations

A strength of this review of reviews is it is the most extensive summary of systematic reviews on the topic to date, as far as we are aware, by including 76 review papers. This research provides the most up-to-date information from recent systematic reviews on key aspects of tobacco and nicotine use in pregnancy. Since smoking in pregnancy is a widely researched topic, this overview summarizes the broad issues and current knowledge while directing readers to more detailed systematic reviews included in this overview [103]. 

A limitation of this review of reviews is that fine details and nuances of the results of primary studies may have been lost [103], for which the reader may need to refer to the component systematic reviews. We also did not perform a quality assessment of the constituent systematic reviews although the inclusion criteria ensured that only relevant systematic reviews were included. We only included reviews published in English, and hence, the generalization of results to a broader range of non-English speaking countries is limited.

## 5. Conclusions

Smoking in pregnancy contributes to a large number of adverse outcomes for both the mother and baby, not only at birth but through the entire life course. NRT and psychosocial interventions (with maximum effect seen for incentive-based interventions) are effective for smoking cessation among pregnant women. Hence, all women who smoke during pregnancy should be provided with evidence-based behavioral support, with the addition of pharmacological support (NRT) if necessary. However, barriers at individual, social and health system levels may reduce the provision and/or patient uptake of smoking cessation therapies during pregnancy. Pregnant women, in general, require broader redress of socio-economic disadvantages and supportive relationships with healthcare providers in order to quit smoking. Healthcare providers require improved behavioral counselling and prescriber training, in combination with conducive workplace systems, to deliver evidence-based multicomponent smoking cessation care for pregnant women.

## Figures and Tables

**Table 1:** Risks of smoking for pregnant women.

Study Number	Author Date	Included Studies	Outcomes Measured	Overall Results
Risks of smoking for pregnant women				
1	Shobeiri (2017) [11]	27	Placental abruption	Based on OR estimates obtained from case–control and cohort studies, there was a significant association between smoking and the risk of placental abruption (OR 1.80; 95% CI: 1.75–1.85; I^2^ = 78.1%, *p* < 0.001). Based on the results of cohort studies, smoking and placental abruption were significantly associated (RR 1.65; 95% CI: 1.51–1.80; I^2^ = 67.1%, *p* = 0.028).
2	Shobeiri (2017) [12]	21	Placenta previa	Based on the random effects model, compared to non-smokers, the estimated OR and RR of placenta previa among smokers was (OR 1.42, 95% CI: 1.30–1.54; I^2^ = 62.7%, *p* < 0.001) and (RR 1.27, 95% CI: 1.18–1.35; I^2^ = 34.6%, *p* = 0.205), respectively.
3	Jenabi (2017) [13]	12	Hyperemesis gravidarum	Compared to non-smokers, the OR of hyperemesis gravidarum among smokers was 0.40 (95% CI: 0.24–0.56; I^2^ = 93.5%, *p* < 0.001).
4	Damron (2017) [14]	24	Relationships among smoking and stress	Significant positive association between measures of stress (measured via subjective self- report measures, open responses in interviews and hair cortisol concentration) or the existence of stressors and the presence of smoking behaviors.
5	Tuenter (2018) [15]	32	levels of folate, Vitamin B12 and homocysteine	Smoking during pregnancy is associated with lower folate and vitamin B12 levels and higher homocysteine levels.
6	Budani (2018) [16]	26	Live birth rate per IVF cycle, clinical pregnancy rate, spontaneous miscarriage	Significant among women who smoke were a decrease in live birth rate per cycle (OR 0.59, 95% CI 0.44–0.79; I^2^ = 30.81%), a lower clinical pregnancy rate per cycle (OR 0.53, 95% CI 0.41–0.68; I^2^ = 49.75%), and an increase in terms of spontaneous miscarriage rate (OR 2.22, 95% CI 1.10–4.48; I^2^ = 53.89%).
7	Purewal (2019) [17]	77 (overall); 28 for smoking	Live births and pregnancy	Women not smoking were significantly more likely to achieve a live birth or pregnancy than those who smoke (OR 1.457, 95% CI: 1.228–1.727, z = 4.324; I^2^ = 51.883; *p* = 0.001).

**Table 2:** Risks of maternal smoking for foetus or child below or equal to two years of age.

Study Number	Author Date	Included Studies	Outcomes Measured	Overall Results
1	Antonopoulos (2011) [18]	12	(i) non-Hodgkin lymphoma (NHL), (ii) Hodgkin lymphoma (HL) and (iii) any lymphoma category in children	Positive association between maternal smoking (any vs. none) during pregnancy and risk for childhood NHL (OR 1.22, 95% CI = 1.03–1.45, fixed effects model; I^2^ = 2.7%, *p* = 0.41). No association found for HL and any childhood lymphoma.
2	Burke (2011) [19]	71	wheeze and asthma in children	Maternal prenatal smoking: increase in risk of wheeze OR = 1.41, 95% CI = 1.20–1.67; I^2^ = 82.5 %), and asthma in children aged ≤2 years (OR = 1.85, 95% CI = 1.35–2.53; I^2^ = 41.9%)
3	Hackshaw (2011) [20]	177	Birth defects in children	Overall odds of birth defects in children: OR = 1.01, 95%CI = 0.96-1.07) Maternal smoking associated with a significant increased risk for defects of the cardiovascular (OR 1.09, 95% CI = 1.02–1.17), musculoskeletal (OR 1.16, 95% CI 1.05–1.27), CNS (OR = 1.10, 95% CI = 1.01–1.19) and gastrointestinal systems (OR 1.27, 95% CI 1.18–1.36; I^2^ = 36%, *P* = 0.02), the face (OR 1.19, 95% CI 1.06–1.35,) including orofacial clefts (OR = 1.28, 95% CI = 1.20–1.36), and cryptorchidism (OR = 1.13, 95% CI = 1.02–1.25). There appears to be a decreased risk for hypospadias and skin defects among babies born to women who smoked.
4	Zhang (2013) [5]	35	Sudden infant death syndrome (SIDS) risk with both prenatal and postnatal maternal smoking.	Prenatal and postnatal maternal smoking was associated with a significantly increased risk of SIDS for prenatal maternal smoking (OR = 2.25, 95% CI = 2.03–2.50, I^2^ = 76.6%, *p* < 0.001), and for postnatal maternal smoking (OR = 1.97, 95% CI = 1.77–2.19; I^2^ = 56.4%, *p* = 0.002) by random effects model.
5	Lee (2013) [21]	35	Congenital Heart Disease (CHD) and CHD subtypes.	Maternal smoking during pregnancy increases the risk of CHDs as a group (RR, 1.11; 95 % CI, 1.02–1.21). There was evidence of heterogeneity across studies (*P* < 0.001) for CHDs overall. Positive associations ranged from 1.02 (fixed effects) for double-outlet right ventricle (95 % CI, 0.72–1.46; n cases = 179) to 1.44 (random effects) for septal defects as a group (95 % CI, 1.16–1.79; n cases = 2977).
6	Nicoletti (2014) [22]	188	Birth defects including cardiovascular, digestive, musculoskeletal, and face and neck	Children of smoking mother had a higher chance of presenting any type of birth defects (OR = 1.18; 95%CI = 1.14–1.22; I^2^ = 77.2%). Significant positive associations between maternal smoking and birth defects in the following body systems: cardiovascular (OR: 1.11; 95%CI: 1.03–1.19), digestive (OR: 1.18; 95%CI: 1.07–1.30), musculoskeletal (OR: 1.27; 95%CI: 1.16–1.39) and face and neck (OR: 1.28; 95%CI: 1.19–1.37).
7	Wang (2014) [23]	13	Neural tube defects (NTDs)	The pooled OR of NTDs in offspring was 1.03 (95%CI = 0.80–1.33; I^2^ = 73.2%, *p* < 0.001) for maternal smoking during pregnancy. The overall effect was 1.55 (95 % CI = 1.06–2.26; I^2^ = 0.0%, *p* = 0.610) for NTDs subtype of spina bifida; the overall effect was 0.94 (95 % CI = 0.71–1.26; I^2^ = 79.2%, *p* < 0.001) for all NTDs subtypes together.
8	Fernandes (2015) [24]	24	Visual outcomes	Most studies (*n* = 18) reported foetal exposure to active or passive maternal cigarette smoking to be associated with an increased risk of adverse visual outcomes in children. In particular, higher rates of strabismus, refractive errors and retinopathy among children of women who smoked during pregnancy.
9	Marufu (2015) [4]	34	Stillbirth	Smoking during pregnancy was significantly associated with a 47% increase in the odds of stillbirth (OR 1.47, 95% CI 1.37–1.57; I^2^ = 79%).
10	Pearson (2015) [25]	8 studies examined tobacco effect	Child cortisol secretion	Maternal smoking acts as a foetal programming factor that increases cortisol secretion in early childhood. The studies that examined prenatal smoking had a combined effect of (d = 0.21, *p* < 0.001, k = 17; Q = 6.04, *p* < 0.05).
11	Silvestri (2015) [26]	43	Asthma or wheezing in offspring of women who smoke during pregnancy	The pooled estimate of the effect of prenatal smoking on current wheezing was OR: 1.36 (95% CI: 1.19–1.55; I^2^ = 68.9%, *p* < 0.001).
12	Tang (2015) [27]	14	Autism Spectrum Disorder (ASD)	The pooled OR was 1.02 (95% CI: 0.93–1.13; I^2^ = 67.3%, *p* < 0.001) comparing mothers who smoked during pregnancy with those who did not.
13	Zhang (2015) [28]	32	Cryptorchidism	The meta-analysis showed that maternal smoking (OR: 1.17, 95% CI: 1.11–1.23; I^2^ = 28.30%, *p* = 0.10) during pregnancy was associated with increased risk of cryptorchidism.
14	Xuan (2016) [29]	29	Oro-facial clefts in children of women who smoke during pregnancy	The overall OR for oro-facial clefts was 1.39 (95% CI = 1.258–1.556; I^2^ = 53.1, *p* = 0.19). A modest but statistically significant association was found between maternal active smoking and cleft lip +/- palate (OR: 1.368; 95% CI: 1.259–1.486; I^2^ = 38.9%, *p* = 0.039) as well as cleft palate (OR 1.241; 95% CI 1.117–1.378; I^2^ = 35.1%, *p* = 0.066).
15	Pineles (2016) [30]	142	Stillbirths, neonatal death and perinatal death	Any active maternal smoking was associated with increased risks of stillbirth (summary relative risk (sRR) = 1.46, 95% CI: 1.38–1.54; I^2^ = 67%, *P* < 0.0001), neonatal death (sRR = 1.22, 95% CI: 1.14–1.30; I^2^ = 39%, *P* < 0.05), and perinatal death (sRR = 1.33, 95% CI: 1.25, 1.41; I^2^ = 60%, *P* < 0.0001). The risks of stillbirth, neonatal death, and perinatal death increased with the amount smoked by the mother.
16	Yan (2016) [31]	49	Acute lymphoblastic leukaemia (ALL)	The pooled ORs showed that there were associations between smoking and Acute lymphoblastic leukaemia (ALL): (Ever vs never, OR: 1.10, 95%CI = 1.02–1.19; I^2^ = 32.7%, *p* = 0.074).
17	Zhang (2017) [32]	43	Congenital heart defects (CHDs) among offspring of maternal smokers.	The pooled RR of any CHD was 1.11 (95% CI: 1.04, 1.18; I^2^ = 69.0%, *p* < 0.001).
18	Pereira (2017) [33]	34	Low birth weight among infants	Active maternal smoking was associated with low birth weight, OR: 2.00 (95% CI: 1.77–2.26; I^2^ = 66.3%).
19	Abraham (2017) [34]	16	Associations between maternal smoking during pregnancy and ultrasound measurements of foetal size	Maternal smoking was associated with reduced second trimester head size (mean reduction 0.09 SD [95% CI: 0.01, 0.16] I^2^ = 56%, *p* = 0.03) and femur length (0.06 [95% CI: 0.01, 0.10] I^2^ = 39%, *p* = 0.13) and reduced third trimester head size (0.18 SD [95% CI: 0.13, 0.23] I^2^ = 22%, *p* = 0.27), femur length (0.27 SD [95% CI: 0.21, 0.32] I^2^ = 30%, *p* = 0.22) and estimated foetal weight (0.18 SD [95% CI: 0.11, 0.24] I^2^ = 50%, *p* = 0.11). Foetal measurements were not reduced for those whose mothers quit before or after becoming pregnant compared to mothers who had never smoked.
20	Koning (2017) [35]	15 (overall); 4 (smoking)	Transcerebellar diameter (TCD) and cellular outcomes in cerebellum	TCD is reduced in smoking compared to non-smoking mothers. Abnormal cytology and increased cell death in offspring of smoking mothers along with increased expression of nicotinic and muscarinic receptors
21	Meng (2018) [36]	23	Neural tube defects (NTDs)	The pooled OR for the risk of NTDs was 1.052 (95% CI = 0.907–1.220; I^2^ = 57.6%, *P* = 0.001) with maternal smoking
22	Quelhas (2018) [37]	201	Small for gestational age (SGA), length/height, and/or head circumference.	Active tobacco use during pregnancy associated with significantly higher rates of SGA (pooled adjusted odds ratio [AORs] = 1.95; 95% CI: 1.76–2.16; I^2^ = 99.2%, *p* < 0.001), shorter length (pooled weighted mean difference [WMD] = 0.43; 95% CI: 0.41–0.44; I^2^ = 93.9%, *p* < 0.001), and smaller head circumference (pooled WMD = 0.27; 95% CI: 0.25–0.29; I^2^ = 90.1%, *p* < 0.001) at birth.
23	Muller-Schulte (2018) [38]	14	Neuroblastoma	Meta-analysis of unadjusted estimates showed an association between tobacco (pooled OR: 1.22; 95% CI 1.04–1.44; I^2^ = 33%) and risk of neuroblastoma during childhood.
24	Palma-Gudiel (2018) [39]	39	DNA methylation, global methylation	Marked tendency towards placental hypomethylation in studies assessing tobacco use during pregnancy. Smoking during pregnancy seems to be associated with widespread hypomethylation.
25	Yu (2019) [40]	20	Cryptoorchidism	The risk of having a male with cryptorchidism significantly increased in women who smoked during pregnancy (pooled crude OR 1.18, 95% CI: 1.12–1.24; I^2^ = 30%, *p* = 0.10).
26	Veisani (2019) [3]	16	Effect of smoking cessation on low birth weight (LBW) and standardized mean differences between smoking cessation intervention and control groups	Incidence of LBW was decreased in the intervention group. The effect of smoking cessation on LBW was OR 0.65, (95% CI: 0.42–0.88; I^2^ = 80.7%; p ≤ 001).

OR—odds ratio; RR—relative risk; CI—confidence interval; SD—standard deviation; d = effect size; AOR—adjusted odds ratio; sRR—summary relative risk; NHL—non-Hodgkin’s lymphoma; HL—Hodgkin’s lymphoma; SIDS—sudden infant death syndrome; CHD—congenital heart disease; NTD—neural tube defect; ASD—autism spectrum disorder; ALL—acute lymphoblastic leukaemia; TCD—transcerebellar diameter; SGA—small for gestational age; LBW—low birth weight.

**Table 3:** Effects of other tobacco products exposure.

Study Number	Author/Date	Included Studies	Outcomes Measured	Overall Results
Effects of exposure to second-hand smoke (SHS) / environmental tobacco smoke (ETS)				
**1**	Salmasi (2010) [42]	76	Primary outcome: perinatal mortality. Secondary outcomes were birthweight, gestational age at delivery, preterm delivery (< 37 weeks gestation), and low birthweight (LBW, < 2,500 g).	No study examined the primary outcome of perinatal mortality. ETS-exposed infants weighed less [WMD –60 g, 95% CI –80 to –39 g; I^2^ = 100%; *p* < 0.00001], with increased risk of low birthweight (LBW, < 2,500 g; RR 1.16; 95% CI 0.99–1.36; I^2^ = 39%, *p* = 0.11), although the duration of gestation and preterm delivery were similar (WMD 0.02 weeks, 95% CI = –0.09 to 0.12 weeks; I^2^ = 60%, *p* = 0.0007 and RR 1.07; 95% CI 0.93–1.22). ETS-exposed infants had longer lengths (WMD = 1.75 cm; 95% CI 1.37–2.12 cm), increased risks of congenital anomalies (OR 1.17; 95% CI 1.03–1.34) and a trend towards smaller head circumferences (WMD = –0.11 cm; 95% CI –0.22 to 0.01 cm).
**2**	Leonardi-Bee (2011) [48]	19	Spontaneous abortion, perinatal and neonatal death, stillbirth, and congenital malformations.	No evidence of a statistically significant effect of SHS exposure on the risk of spontaneous abortion (OR: 1.17 [95% CI: 0.88–1.54; I^2^ = 66%, *p* = 0.008]. SHS exposure significantly increased the risk of stillbirth (OR: 1.23 [95% CI: 1.09–1.38; I^2^ = 0%, *p* = 0.60]; and congenital malformation (OR: 1.13 [95% CI: 1.01–1.26; I^2^ = 18%, *p* = 0.30]; 7 studies), SHS had no significant effect on perinatal or neonatal death.
**3**	Burke * (2011) [19]	71	Wheeze and asthma during 3 different age ranges (≤2 years, 3 to 4 years, 5 to 18 years).	Exposure to postnatal maternal smoking was associated with the strongest effects on the incidence of wheeze, ≤2 years (OR 1.70, 95% CI 1.24–2.35, I² = 0%). Passive household smoking: increased the risk of wheeze in children aged ≤2 years (OR = 1.35, 95% CI = 1.10–1.64, I² = 64.5%, 9 studies).
**4**	Jones (2011) [49]	60	Lower respiratory infections (LRI), with diagnostic subcategories including bronchiolitis, in infants aged two years and under.	Exposure to smoking by any household member was associated with a statistically significant increase in the odds of LRI for infants <2 years by 1.54 (95% CI 1.40 to 1.69; I^2^ = 62%, *p* < 0.00001). Both parents smoking demonstrated a statistically significant increase in the odds of LRI, by 1.62 (95% CI 1.38 to 1.89; I^2^ = 65%, *p* = 0.0004) Maternal smoking after birth was associated with a statistically significant increase in odds of LRI, by 1.58 (95% CI 1.45 to 1.73; I^2^ = 57%, *p* < 0.0001.
**5**	Tsai (2017) [45]	16	Children’s health outcomes.	ETS may affect infant birth weight, children’s neurodevelopment, and development of allergies
**6**	Suzuki (2019) [50]	8	Initiation of breastfeeding. Exclusive or partial breastfeeding was measured as prevalence or duration. Discontinuation of breastfeeding 6 months after birth.	There was a significant increased risk of discontinuation of any breastfeeding before six months for women who were exposed to SHS during pregnancy (pooled OR = 1.07 [95% CI: 1.01–1.14; I^2^ = 34%)
**7**	Suzuki (2019) [53]	7	Depressive symptoms during pregnancy and postpartum in pregnant women exposed to SHS	Depressive symptoms at any time during pregnancy and postpartum significantly increased (OR = 1.77 [95% CI = 1.12–2.79]; I^2^ = 28%, *n* = 4103, two studies). Increased odds of antenatal suicidal ideation in SHS exposed women (OR = 1.75 [95% CI = 1.14–2.70; I^2^ = 51%, *n* = 2670, two studies).
**8**	Sabbagh (2015) [51]	15	Non syndromic orofacial clefts (NSOFC) in offspring of women exposed to SHS	There was a significant relationship between passive maternal smoking and NSOFC. (OR: 2.11, 95% CI: 1.54 to 2.89; I^2^ = 91%, *p* < 0.00001).
**9**	Silvestri * (2015) [26]	43	Asthma or wheezing in offspring who are exposed to smoke after birth	Association between postnatal maternal smoking and wheezing in the past 12 months had an effect size of 1.21 (95% CI: 1.13–1.31; I^2^ = 47.0%, *p* = 0.067). The pooled estimate of the effect of postnatal exposure to parental smoking was very similar to that of exposure to maternal smoking: OR: 1.30 (95% CI: 1.13–1.51; I^2^ = 71.1%, *p* = 0.002).
**10**	Cui (2016) [52]	24	Preterm birth in offspring of women exposed to SHS during pregnancy	Overall, the SORs of preterm birth for women who were ever exposed to passive smoking versus women who had never been exposed to passive smoking at any place and at home were 1.20 (95%CI = 1.07–1.34, I^2^ = 36.1%) and 1.16 (95%CI = 1.04–1.30, I^2^ = 4.4%), respectively.
**11**	Meng * (2018) [36]	23	Neural tube defects (NTDs)	The pooled OR for the risk of NTDs 1.898 (95% CI 1.557–2.313; I^2^ = 50.5%) with passive smoking.
Effects of smokeless tobacco products exposure				
**1**	Ratsch (2014) [54]	21	(1) Birth outcome (live/stillbirth), (2) foetal distress, neonatal apnoea, early neonatal death and neurobehavioural assessment, (3) gender ratio, (4) gestational age and (5) anthropometric measures.	Many studies lacked sufficient power to estimate precise risks. However, there were indications that maternal smokeless tobacco use increases rates of stillbirth, low birth weight and alters the male: female live birth ratio.
**2**	Inamdar (2015) [44]	9 Observational studies (16 reports)	Adverse health outcomes in newborns including LBW, preterm, stillbirth and SGA,	Significant associations with ST use were seen in for LBW, preterm, stillbirth and SGA. Heterogeneity between results was moderate for LBW (I^2^ = 44%) and stillbirth (I^2^ = 52%), and high for preterm (I^2^ = 87%) and SGA (I^2^ = 65%). Meta-analysis was not undertaken.
**3**	Suliankatchi (2016) [43]	2	Low birth weight, pre-term birth and still birth in offspring of women who use ST during pregnancy	Pooled odds ratio was significant for all three outcomes: low birth weight (OR 1.88, 95 % CI 1.38–2.54; I^2^ = 38 %), preterm birth (OR 1.39, 95 % CI 1.01–1.91; I^2^ = 0%) and stillbirth (OR 2.85, 95 % CI 1.62–5.01; I^2^ = 0%).
Effects of water pipe smoking				
**1**	El-Zaatari (2015) [46]	49	Obstetrical and perinatal outcomes	Water pipe smoking (WPS) has been associated with obstetric and perinatal complications including low birthweight (LBW), infant mortality, low APGAR scores, and pulmonary complications at birth. Three studies reported an overall 2.12 times odds of LBW in association with WPS.
**2**	Akl (2011) [55]	3	Pregnancy outcomes (low birth weight) and infertility	Water pipe tobacco smoking was associated with low birth weight (OR = 2.12; 95% CI 1.08–4.18; I^2^ = 0%, *p* = 0.55) and infertility (OR = 2.5; 95% CI 1.0–6.3).

OR—odds ratio; RR—relative risk; CI—confidence interval; SD—standard Deviation; d = effect size; AOR—adjusted odds ratio; SOR—summary odds ratio; ETS—environmental tobacco smoke; LBW—low birth weight; WMD—weighted mean difference; ST—smokeless tobacco; APGAR score: appearance, pulse, grimace, activity, and respiration; NSOFC—non syndromic orofacial clefts; SGA—small for gestational age; LRI—lower respiratory tract infection; SHS—second-hand smoke. * Studies examining both the effects of smoking in pregnancy as well as other tobacco products or secondhand smoke exposures.

**Table 4:** Interventions directed smoking cessation and reducing other tobacco products or secondhand smoke exposure during pregnancy.

Study Number	Author/Date	Included Studies	Interventions	Outcomes	Results
Interventions directed at smoking cessation					
**1**	Akerman (2015) [56]	Three trials of any study type and design evaluating any treatment for smoking in pregnant women undergoing opioid medication-assisted treatment	One trial used contingency management (incentive-based treatment), two trials used brief behavioral interventions	Daily self-reported cigarette use in the pregnant methadone-maintained women, carbon monoxide and cotinine levels	Contingency management/ incentive based treatment, was the most promising intervention: 31% of participants achieved abstinence within the 12-week study period, compared to 0% in a non-contingent behavior incentive group and a group receiving usual care. Two studies of brief behavioral interventions resulted in reductions in smoking but not cessation.
**2**	Filion (2011) [57]	Eight RCTs conducted in pregnant women in which the effect of counselling could be isolated. Trials reported biochemically validated abstinence at 6 or 12 months after the target quit date.	Counselling, including minimal clinical intervention, individual counselling, group counselling or telephone counselling	Abstinence at 6 months. Measures were biochemically validated using expired carbon monoxide or salivary cotinine.	The proportion of women that remained abstinent at the end of follow-up was modest, 4 to 24% among those randomized to counselling and from 2 to 21% among control women. The absolute difference in abstinence reached a maximum of only 4%. Summary estimates are inconclusive because of wide confidence intervals, albeit with little evidence to suggest that counselling is efficacious at promoting abstinence (OR 1.08, 95% CI 0.84–1.40; I^2^ = 0%)
**3**	Myung (2012) [58]	Seven (five RCTs, one quasi-RCT and one prospective study	Pharmacotherapy (NRT and Bupropion)	Smoking cessation (assessed by both self-report and biochemical verification)	In a fixed-effects meta-analysis of all seven studies based on the longest follow-up data available, pharmacotherapy had a significant effect on smoking cessation (relative risk RR = 1.80; 95% CI = 1.32–2.44; I^2^ = 41.5%). The abstinence rate at late pregnancy in the intervention ranged from 7% to 22.6% (mean abstinence rate 13.0%; 95% CI 10.9–15.2%; Cochrane’s Q = 0.062). Effect was strongest for midterm (12–24 weeks) follow-up (RR 1.65, 95% CI 1.20–2.28; I^2^ = 46.7%) and least for long term (>24 weeks) follow-up studies (RR 1.34, 95% CI 0.90–1.99 I^2^ = 0%).
**4**	Hemsing (2012) [59]	Nine interventional studies.	Interventions to enhance partner support for pregnant/postpartum women’s smoking reduction or cessation and cessation treatments for the partners themselves. For example, quit smoking counselling/resources to pregnant women and/or their partners, a mass media campaign, biofeedback interventions, and providing information booklets aimed at facilitating partner support.	Smoking cessation of a pregnant women and/or partner	Very few intervention studies demonstrated significant results in either encouraging partners to support smoking cessation during pregnancy and postpartum or in improving the partner’s smoking cessation. Overall, there is limited evidence for the efficacy of encouraging partners to support smoking cessation during pregnancy and postpartum.
**5**	Chamberlain (2017) [60]	A total of 102 randomized controlled trials, cluster-randomized trials, and quasi-randomized controlled trials of psychosocial smoking cessation interventions during pregnancy	Psychosocial interventions: counselling, health education, feedback, incentives, social support, exercise and dissemination	smoking abstinence	High quality evidence that counselling increased smoking cessation in late pregnancy compared with usual care (RR = 1.44, 95% CI = 1.19–1.73; I^2^ = 49%) and less intensive interventions (RR = 1.25, 95% CI 1.07–1.47; I^2^ = 28%). High-quality evidence suggests incentive-based interventions are effective when compared with an alternative (non-contingent incentive) intervention (RR 2.36, 95% CI = 1.36–4.09; I^2^ = 0%). High-quality evidence suggests the effect is unclear in social support interventions provided by peers (RR 1.42, 95% CI 0.98–2.07). High quality evidence from pooled results demonstrated that women who received psychosocial interventions had a reduction in adverse birth outcomes.
**6**	Passey (2013) [61]	Two interventional studies with control group	Culturally tailored interventions for Indigenous populations. These used face-to-face counselling, structured follow-up, family involvement and nicotine replacement therapy (NRT).	Smoking cessation among pregnant Indigenous women	Both studies found no treatment effect. The systematic review found that there is currently no evidence for interventions that are effective in supporting pregnant Aboriginal and Torres Strait Islander women to quit smoking.
**7**	Coleman (2015) [64]	Nine RCTs on the efficacy of pharmacotherapies for smoking cessation in pregnancy	Pharmacotherapy (Nicotine Replacement Therapy (NRT) or Bupropion)	Primary efficacy outcome was smoking cessation in later pregnancy (in all but one trial, at or around delivery); safety included 11 outcomes (principally birth outcomes) related to neonatal and infant well-being	Compared to placebo and non-placebo controls, there was a difference in smoking rates observed in later pregnancy favoring use of NRT (risk ratio (RR) 1.41, 95% CI = 1.03 to 1.93; I^2^ = 18%). In the one trial of bupropion (Stotts 2015), two (out of five) placebo group participants had validated smoking cessation, but no bupropion group participants reported abstinence.
**8**	Jones (2015) [47]	A total of 18 studies of interventions delivered to pregnant women, which reported any relevant economic evaluation metric.	Any interventions or combination of interventions, both real and hypothetical (an intervention with an assumed quit rate for economic modelling), aimed at encouraging pregnant smokers to quit. Interventions included counselling, self-help materials, NRT, financial incentives and physical activity.	Clinical or economic outcomes considered relevant to the mother and/or child (e.g., smoking status at end of pregnancy, low birth weight (LBW) (birth weight < 2500 g) births averted, sudden infant deaths (SIDs) averted, and quality adjusted life years (QALYs).	Seventeen studies identified that within-pregnancy interventions are cost-effective, with only one trial reporting that usual care was better than the experimental intervention (motivational interviewing)
**9**	Washio (2016) [62]	Nine controlled studies of predominantly racial/ethnic-minority pregnant smokers	Most studies provided some form of brief smoking cessation counselling, with two adding incentives and one adding pharmacotherapy.	Biochemically-verified smoking abstinence with breath, saliva, or urine samples and/or self-reported smoking abstinence. Birth outcomes were also reported.	Treatment effects on the smoking outcomes were not consistently significant among the reviewed studies. Three studies provided biochemically-verified outcomes, showing high postpartum relapse rates. Reduction in smoking during pregnancy was reported in three studies defined as a fifty percent decrease in cotinine levels from baseline to the end of pregnancy or as decrease in the number of cigarettes smoked per day during pregnancy. Not all reports showed significant smoking reduction.
**10**	Arden-Close (2017) [63]	A total of 14 studies (overall); 2 studies for smoking in pregnancy	Couple based counselling interventions for smoking cessation in pregnant women	smoking cessation	A non-randomized intervention study (Øien et al., 2008) of three min of advice given to expectant couples by a health care professional during an antenatal appointment did not influence smoking cessation six weeks post-birth. Similarly, an RCT of a couple-based intervention (six counselling calls; three during pregnancy, three post-partum) supplemented by a booklet and video did not increase smoking cessation at 12 months post-partum relative to usual care (McBride et al., 2004).
Interventions directed at other tobacco products use or second-hand smoke exposure					
**11**	Duckworth (2012) [65]	A total of 5 original research reports of smoking cessation interventions for partners of pregnant or postpartum women through 12 months after delivery	Interventions included telephone support, couple support and communication, nicotine patches, and various modes of cessation education.	Quit rates of partners of women who are pregnant	Four of the studies yielded significantly reduced post-intervention smoking rates among the partners. One intervention had no effect on the partners’ smoking.
**12**	Tong (2015) [66]	A total of 5 randomized controlled trials which met the inclusion criteria: non-smoking pregnant women exposed to SHS, clinical interventions that intended to reduce SHS, a control group and outcomes included reduction in SHS or quit rates among partners	Four of the studies involved psychosocial interventions delivered to pregnant women within the antenatal care setting, and the fifth study involved psychosocial intervention plus medication to partners of pregnant women.	Pregnant women’s exposure to second-hand smoke (SHS) and quit rates among partners of pregnant women	Results from all five studies showed positive findings based on study-defined outcome measures. Four of the studies showed reduced exposure in pregnant women and one study reported 7- and 30-day abstinence in partners of pregnant women.
**13**	Dherani (2017) [67]	Six clinical trials. Participants were men encouraged to change their smoking behaviors by their pregnant wife/partner.	Behavior change interventions (BCI) to reduce SHS at home, compared to no intervention or usual care.	Self-reported or objectively assessed (nicotine/cotinine/ CO levels or clinical measures) SHS exposure of the pregnant woman at home; smoking behavior of the man, or awareness/knowledge of the risks of SHS.	The BCI administered showed a low to moderate success in achieving the selected outcomes.

OR—odds ratio; RR—relative risk; CI—confidence interval; SD—standard deviation; QALYs—quality adjusted life years; LBW—low birth weight; BCI—behavior change interventions.

**Table 5:** Barriers and predictors of smoking cessation among pregnant women.

Study Number	Author/Date	Included Papers	Population/Outcomes Assessed	Results
**1**	Baxter (2010) [68]	23; 10 qualitative, 10 quantitative (cross sectional data (surveys)) and 3 narrative.	All women who smoke who are planning a pregnancy, are pregnant, or have an infant aged less than 12 months. The review examined factors underpinning the delivery of interventions to this population from the perspective of staff, users, and potential service users.	Key themes included: 1. Whether or not a health professional mentions smoking 2. The content of advice and information provided 3. The manner of communication 4. Use of service protocols 5. Follow-up discussion 6. Staff confidence in their skills 7. The impact of time and resource constraints 8. Staff perceptions of ineffectiveness 9. Differences between professionals 10. Obstacles to accessing interventions.
**2**	Ingall (2010) [69]	7. Only qualitative studies that collected data during the postpartum stage about changes made to smoking behavior during pregnancy.	Women (15 years or over) who had attempted to quit smoking during pregnancy.	Women’s awareness about health risks to the foetus was not sufficient motivation to quit. Barriers to quitting included: willpower, role and meaning of smoking, issues with cessation provision, changes in relationship interactions, understanding of facts, changes in smell and taste and influence of family and friends. Cessation service provision by health professionals was viewed negatively by women.
**3**	Okoli (2010) [70]	28 quantitative articles on assessments of and interventions addressing health care providers’ (HCP) delivery of care among pregnant girls and women.	HCP with pregnant clients	Although > 50% of health care practitioners are likely to ask women about their smoking status and advise pregnant smokers to quit, <50% assess readiness to change, assist in smoking cessation, or arrange for follow-up appointments/referrals. Important provider-specific, patient-specific, and system/organizational barriers were found to hinder the provision of smoking cessation care by the health care practitioner.
**4**	Schneider (2010) [82]	19	Characteristics of pregnant women who quit and who continue to smoke during pregnancy	Predictors of smoking during pregnancy were: a partner who smokes, a large number of children, a high rate of tobacco consumption and deficiencies in prenatal care.
**5**	Gould (2013) [71]	7; 4 qualitative (focus groups) and 3 quantitative (questionnaires)	Aboriginal and Torres Strait Islander women. Outcomes assessed were experiences of smoking, experiences of environmental tobacco smoke (ETS), knowledge of health effects of smoking and ETS, beliefs about and attitudes to the health effects of smoking and ETS, knowledge about cessation, beliefs and attitudes about cessation, strategies for cessation, and influences on and barriers to cessation.	A total of eleven third-order constructs operating on the levels of self, family, and social networks, the wider Aboriginal community and broader external influences. Highlighted are social norms and stressors within the Aboriginal community perpetuating tobacco use; insufficient knowledge of smoking harms; inadequate saliency of antismoking messages; and lack of awareness and use of pharmacotherapy. Indigenous health workers have a challenging role, not yet fulfilling its potential. Pregnancy is an opportunity to encourage positive change where a sense of a “protector role” is expressed.
**6**	Crane (2013) [72]	31	Strength of relationship between smoking and intimate partner violence (IPV) among pregnant women	Women who have experienced IPV are at greater risk of smoking than those who have not. Subsequent moderator analyses indicated that the association is moderately stronger among pregnant compared to non-pregnant victims. The strength of the victimization-smoking relationship did not differ by relationship type or ethnicity.
**7**	Flemming (2013) [73]	*N* = 29 (26 studies)	Pregnant women who were smokers prior to pregnancy and who attempted to quit or continued to smoke during pregnancy. A synthesis of women’s experiences influencing their smoking behavior in pregnancy, including attempts to quit, used meta-ethnography.	Four lines of argument were identified to trace the journeys made by women who were smokers at the start of their pregnancy namely: 1) being a smoker, 2) being a pregnant smoker, 3) quitting and trying to quit smoking, and 4) continuing to smoke. Important themes were: the embeddedness of smoking in women’s lives, questioned only because of pregnancy; quitting for pregnancy rather than for good; quitting had significant costs for the woman and cutting down was a positive alternative; the role of partners and the influence of relationship dynamics on women’s smoking habits
**8**	Bottorf (2014) [74]	40 (39 quantitative and 1 qualitative)	Adolescents aged 19 and under who used alcohol and tobacco during pregnancy and the postpartum period. Outcomes included identifying trends and predictors of alcohol and tobacco use, prior to, during and following pregnancy	Six predictors of tobacco use were: degree of nicotine dependence; number of cigarettes smoked in the last month; alcohol intake pre- pregnancy; religiosity; maternal encouragement to quit; and compatibility of peer and parent attitudes. Tobacco use was significantly related to alcohol use; pregnant adolescents who continued to smoke into the third trimester had more friends who smoked, did not live with a parent, engaged in binge drinking in the first trimester, experienced earlier age of first intercourse and were white. Psychological factors predicting higher levels of smoking included: previous childhood physical or sexual abuse, intention to control weight using cigarettes, depression and anxiety.
**9**	Flemming (2015) [75]	42 (38 studies)	Facilitators and barriers to quitting smoking among pregnant women, the majority from disadvantaged groups	Four factors acted both as barriers and facilitators to women’s ability to quit smoking in pregnancy and postpartum: psychological well-being, relationships with significant others, changing connections with her baby through and after pregnancy; appraisal of the risk of smoking.
**10**	Graham (2014) [76]	29 (26 studies)	Exploration of pregnant women’s perceptions and experiences of cutting down.	Cutting down was both a method of quitting and, for persistent smokers, a method of harm reduction. While pregnant women were aware that official advice was to quit abruptly, cutting down was seen as a positive behavior change in often difficult domestic circumstances, and one that health professionals condoned.
**11**	Rhodes-Keefe (2015) [77]	4	Relationship between smoking status, rurality, and depression in the pregnant population.	Smoking has been associated with depression in the rural pregnant population. Depression and limited supports promote continued smoking. Rural women do not necessarily identify themselves as depressed. The role of rurality in depression in pregnant smokers is uncertain.”
**12**	Bauld (2017) [78]	65 (55 studies)	Pregnant women, their partners and health providers. The perceived barriers to, and facilitators of, smoking cessation in pregnancy and the identification of potential new/modified interventions.	Themes central to cessation in pregnancy at an individual level: perception of risk to baby, self-efficacy, influence of close relationships and smoking as a way of coping with stress. Interpersonal level: partners’ emotional and practical support, willingness to change smoking behavior and role of smoking within relationships were important. Important across the review and the interviews of HPs were: education to enhance knowledge and confidence in delivering information about smoking in pregnancy and the centrality of the client relationship, and protection of which could be a factor in downplaying risks.
**13**	Riaz (2018) [79]	55 (observational studies and clinical trials)	Predictors of both biochemically validated and non-biochemically validated smoking abstinence in pregnancy	The most frequently observed significant factors associated with cessation were: higher level of education (OR 2.16, 95% CI: 1.80–2.84; I^2^=93.2%, *p* < 0.001), higher socio-economic status: (OR 1.97 95% CI: 1.20–3.24; I^2^ = 82.3%, *p* = 0.004), overseas maternal birth: (OR 2.00, 95% CI: 1.40–2.84; I^2^ = 81.9%, *p* = 0.001), Medicaid coverage or private insurance: (OR 1.54 95% CI: 1.29–1.85; I^2^ = 41.5%, *p* = 0.145), living with partner or married: (OR 1.49, 95% CI: 1.38–1.61; I^2^ = 60.5%, *p* = 0.019), partner/other members of the household do not smoke: (OR 0.42, 95% CI: 0.35–0.50; I^2^ = 71.9%, *p* < 0.001), lower heaviness of smoking index score: (OR 0.45, 95% CI: 0.27–0.77;I^2^ = 87.5%, *p* < 0.001) lower baseline cotinine level: (OR 0.78, 95% CI: 0.64–0.94; I^2^ = 96.6%, *p* < 0.001), low exposure to second-hand smoking: (OR 0.45, 95% CI: 0.20–1.02; I^2^ = 95.3%, *p* < 0.001), not consuming alcohol before and/or during pregnancy: (OR 2.03, 95% CI: 1.47–2.80;I^2^ = 0.0%, *p* = 0.950), primiparity: (OR 1.85, 95% CI: 1.68–2.05; I^2^ = 78.4%, *p* < 0.001), planned breastfeeding: (OR 1.99 (95% CI: 1.94–2.05;I^2^ = 0.0%, *p* = 0.514), perceived adequate pre-natal care: (OR 1.74, 95% CI: 1.38–2.19;I^2^ = 92.3%, *p* < 0.001), no depression: (OR 2.65, 95% CI: 1.62–4.30; I^2^ = 0.0%, *p* = 0.694) and low stress during pregnancy: (OR 0.58, 95% CI: 0.44–0.77;I^2^ = 0.0%, *p* = 0.887)
**14**	Small (2018) [80]	13	Experiences of smoking during pregnancy for Indigenous women and the smoking cessation needs of Indigenous women during pregnancy.	Being pregnant is a motivator for Indigenous women to quit, try to quit, or cut down on smoking, mainly because they want to protect their children from the harmful effects of maternal smoking during pregnancy, but also because of biological (morning sickness and altered taste and smell for cigarettes) and environmental deterrents (perceived social pressure to quit) to smoking during pregnancy. Barriers to quitting include smoking dependence, being under stress, living in a smoking environment, lacking social support for quitting, rejecting or not knowing the facts about smoking harms, unreceptivity to anti-smoking messages, and boredom.
**15**	Harris (2019) [81]	9	Facilitators and barriers to smoking cessation amongst Aboriginal and/or Torres Strait Islander women during pregnancy.	Social and familial influences and daily stress have a strong impact on whether a woman feels she can quit smoking during pregnancy. Information and advice regarding potential adverse effects of smoking on the foetus, or lack thereof, from HPs either facilitated cessation of smoking in pregnancy or was a barrier to quitting. A lack of awareness from midwives and doctors on smoking cessation strategies, such as nicotine replacement therapy, was a barrier for women

OR—odds ratio; RR—relative risk; CI—confidence interval; IPV—intimate partner violence.
